# Genome-wide DNA methylation status is a predictor of the efficacy of anti-EGFR antibodies in the second-line treatment of metastatic colorectal cancer: Translational research of the EPIC trial

**DOI:** 10.1007/s00384-024-04659-y

**Published:** 2024-06-11

**Authors:** Kota Ouchi, Shin Takahashi, Keiju Sasaki, Yuya Yoshida, Sakura Taniguchi, Yuki Kasahara, Keigo Komine, Hiroo Imai, Ken Saijo, Hidekazu Shirota, Masanobu Takahashi, Chikashi Ishioka

**Affiliations:** 1 Department of Medical Oncology, Tohoku University Hospital, Miyagi, Japan. 4-1 Seiryo-Machi, Aobaku, Sendai, Miyagi 980-8575 Japan; 2 Department of Clinical Oncology, Graduate School of Medicine, Tohoku University, Miyagi, Japan. 4-1 Seiryo-Machi, Aobaku, Sendai, Miyagi 980-8575 Japan; 3 Department of Clinical Oncology, Institute of Development, Aging and Cancer, Tohoku University, Miyagi, Japan 4-1 Seiryo-Machi, Aobaku, Sendai, Miyagi 980-8575 Japan

**Keywords:** Colorectal cancer, Biomarker, Predictive factor, Anti-EGFR antibody, Genome-wide DNA methylation status

## Abstract

**Purpose:**

The genome-wide DNA methylation status (GWMS) predicts of therapeutic response to anti-epidermal growth factor receptor (EGFR) antibodies in treating metastatic colorectal cancer. We verified the significance of GWMS as a predictive factor for the efficacy of anti-EGFR antibodies in the second-line treatment of metastatic colorectal cancer.

**Methods:**

Clinical data were obtained from a prospective trial database, and a genome-wide DNA methylation analysis was performed. GWMS was classified into high-methylated colorectal cancer (HMCC) and low-methylated colorectal cancer (LMCC). The patients were divided into subgroups according to the treatment arm (cetuximab plus irinotecan or irinotecan alone) and GWMS, and the clinical outcomes were compared between the subgroups.

**Results:**

Of the 112 patients, 58 (51.8%) were in the cetuximab plus irinotecan arm, and 54 (48.2%) were in the irinotecan arm; 47 (42.0%) were in the HMCC, and 65 (58.0%) were in the LMCC group regarding GWMS. Compared with the LMCC group, the progression-free survival (PFS) was significantly shortened in the HMCC group in the cetuximab plus irinotecan arm (median 1.4 vs. 4.1 months, p = 0.001, hazard ratio = 2.56), whereas no significant differences were observed in the irinotecan arm. A multivariate analysis showed that GWMS was an independent predictor of PFS and overall survival (OS) in the cetuximab plus irinotecan arm (p = 0.002, p = 0.005, respectively), whereas GWMS did not contribute to either PFS or OS in the irinotecan arm.

**Conclusions:**

GWMS was a predictive factor for the efficacy of anti-EGFR antibodies in the second-line treatment of metastatic colorectal cancer.

**Supplementary Information:**

The online version contains supplementary material available at 10.1007/s00384-024-04659-y.

## Introduction

Molecular biological studies performed over the past several decades have gradually revealed the molecular mechanisms of cancer, leading to the development of molecular targeted therapies. In the context of systemic chemotherapy for metastatic colorectal cancer (mCRC), new molecular targeted agents such as anti-vascular endothelial growth factor antibodies [1], anti-epidermal growth factor receptor (EGFR) antibodies [2, 3], and multi-tyrosine kinase inhibitors [4] were sequentially introduced. Thus, the life expectancy of patients with mCRC was extended by approximately 3 years [5, 6].

Anti-EGFR antibodies, such as cetuximab, exert their antitumor effects by directly binding to EGFR and inhibiting its downstream signaling [2, 3, 7]. Anti-EGFR antibodies have demonstrated clinical efficacy in combination with cytotoxic agents in first-line or second-line treatment and as single agents in third-line or later treatment, and are important molecular targeted agents in the treatment of mCRC [3, 6–9]. Molecular targeted agents are more effective than conventional cytotoxic agents, and their efficacy is further enhanced by biomarkers that are used to identify appropriate patients for treatment [10]. Conversely, the cost-effectiveness of molecular targeted agents is likely an issue because of their high cost [11]. Therefore, the stratification of patients using biomarkers has been used to improve cost-effectiveness issues [12, 13]. In addition, to avoid side effects among patients who are refractory to treatment, it is necessary to apply molecular targeted agents to appropriately selected patients where it will be effective.

As important biomarkers for determining the application of anti-EGFR antibodies, genetic factors such as the *RAS* and *BRAF* genotype are well known and have been used [14–16]. Recently, anatomical factors such as the primary tumor site have been included in several practice guidelines and are used in clinical practice [17, 18].

Aberrant DNA methylation, such as the CpG island methylator phenotype (CIMP), is an important oncogenic mechanism of colorectal cancer [19–21] and has been reported to be associated with molecular biological features [22, 23] and prognosis [24, 25]. We focused on this epigenetic factor and performed a comprehensive DNA methylation analysis of patients who received anti-EGFR antibodies as the third-line or later treatment [26]. We observed a strong correlation between the genome-wide DNA methylation status (GWMS) and the clinical outcomes of anti-EGFR antibodies, and we found that high-methylated colorectal cancer (HMCC) was refractory to anti-EGFR antibodies compared with low-methylated colorectal cancer (LMCC). Furthermore, the GWMS was shown to be a predictor of clinical outcomes and that was independent of *RAS*/*BRAF* mutation status and primary tumor site.

Based on the abovementioned findings, we developed a novel diagnostic method for GWMS evaluation using the MethyLight assay [27]. To verify the predictive accuracy of this assay, retrospective analyses were performed on patients who received anti-EGFR antibodies as first-line, second-line, third-line, or later treatment, respectively [27–29]. The results showed that the GWMS classification (HMCC or LMCC) using the modified MethyLight assay was significantly associated with the therapeutic efficacy of anti-EGFR antibodies in all three studies, suggesting its clinical usefulness. However, these reports only included patients who received anti-EGFR antibodies, with no studies directly comparing clinical outcomes between groups that received anti-EGFR antibodies and those that did not receive them. Therefore, it was difficult to determine whether GWMS was more significant as a predictive factor or as a prognostic factor in the context of anti-EGFR treatment.

The EPIC trial was designed to determine whether the addition of cetuximab to irinotecan as a second-line treatment for mCRC would contribute to prolonged survival [9]. The objective of this translational research was to evaluate the potential of genome-wide DNA methylation status as a predictive biomarker for the effectiveness of anti-EGFR antibodies in the second-line treatment for patients with metastatic colorectal cancer.

## Patients and methods

### Patients

We included patients who were enrolled in the EPIC trial [9] between May 2003 and February 2006 and for whom formalin-fixed, paraffin-embedded (FFPE) specimens of the primary tumor were available. The EPIC trial was a randomized, open-label, phase III study evaluating the add-on effect of cetuximab, an anti-EGFR antibody, to irinotecan monotherapy as a second-line treatment for patients with mCRC. Patients who had received previous irinotecan or anti-EGFR therapies were not eligible to be enrolled in that trial. The detailed eligibility criteria for the EPIC trial were as previously described [9]. The enrolled patients were randomly assigned to the cetuximab plus irinotecan arm (CETU/IRI arm) or the irinotecan alone arm (IRI arm). The patients were assigned to each treatment arm while the significance of *RAS* and *BRAF* genotypes in anti-EGFR treatment was unclear during the planning of the EPIC trial. The study was conducted in accordance with the Declaration of Helsinki. The protocol was approved by the ethics committees of all participating centers, and all patients provided written informed consent. In conducting this translational research, approval was also obtained from the Tohoku University Hospital Ethics Committee (Approval No. 2022–1-737).

Data on progression-free survival (PFS), overall survival (OS), best overall response (BOR), gender, age, primary tumor site, race, Eastern Cooperative Oncology Group Performance Status (ECOG PS), presence of liver metastases at the baseline, and assigned treatment arm were obtained from the EPIC trial database. The patients for whom GWMS could be determined were included in the final analysis.

### Treatment

In the CETU/IRI arm, cetuximab (400 mg/m^2^) was initially administered (2 h intravenously [IV]), followed by 250 mg/m^2^ weekly (1 h IV) and preceded by premedication with antihistamine. Irinotecan (350 mg/m^2^, 300 mg/m^2^ for patients with 90 min IV, age 70 years, ECOG PS 2, and a history of pelvic or abdominal irradiation) was administered every 3 weeks in both treatment arms, with patients in the CETU/IRI arm receiving it 1 h after the completion of the cetuximab infusion.

Treatment was continued until disease progression or unacceptable toxicity was observed. There were no treatment restrictions after the completion of the study, and anti-EGFR antibodies as a post-treatment option were available.

### Outcomes

Tumor response was evaluated every 6 weeks according to the modified WHO criteria. PFS was defined as the period from the date of study enrollment to the date of disease progression, and OS was defined as the period until the date of patient death. The response rate (RR) was calculated by dividing the total number of patients with CR and PR by the total number of patients for whom BOR could be determined.

Clinical outcomes (PFS, OS, and RR) were compared between the two groups with different GWMS (HMCC vs. LMCC) and between the two groups with different treatment arms (CETU/IRI arm vs. IRI arm).

### Extraction of DNA from tumor specimens

DNA was extracted from FFPE surgical specimens of the primary tumor. Tumor specimens were stored at 4 °C until DNA was extracted. Hematoxylin–eosin-stained specimens were used to guide macro-dissection in areas containing cancer cells. Genomic DNA (gDNA) was extracted from the dissected specimens using a QIAamp DNA FFPE Tissue Kit (Qiagen, Hilden, Germany).

### Determination of the genome-wide DNA methylation status

The genome-wide DNA methylation status was measured as previously described [27]. A modified MethyLight assay was performed on 16 cytosine-guanine dinucleotide (CpG) sites to determine whether the tumor was HMCC or LMCC. Of the 16 CpG sites, tumors with eight or more methylation-positive CpG sites were classified as HMCC, and tumors with seven or fewer methylation-positive CpG sites were classified as LMCC.

### Statistical analysis

All statistical analyses were performed using JMP (JMP®, Version 16.0.0. SAS Institute Inc., Cary, NC, USA). One-way ANOVA was used to test continuous variables. Pearson χ2 test was used to compare nominal variables between the two groups, and two-sided Fisher’s exact test was used when more than 20% of the cells had an expected frequency of less than five or at least one cell had an expected frequency of less than 1, for the cross-tabulation table. The Kaplan–Meier method was used for comparative survival analysis and median calculation, and the significance between the two groups was verified using the log-rank test. Univariate and multivariate analyses were performed using the Cox proportional hazards model.

For the statistical tests performed in this study, p < 0.05 was considered significant.

## Results

### Patients

Of the patients assigned to the treatment arms of the EPIC trial (n = 1,298), 112 patients with a measurable GWMS were included in the analysis (Supplementary Fig. 1). Of these, 58 (51.8%) were in the CETU/IRI arm, and 54 (48.2%) were in the IRI arm. The primary tumor sites were the colon in 97 patients (86.7%) and the rectum in 15 patients (13.3%) (Table 1).

**Table 1:** Patient characteristics in each treatment arm and GWMS

Characteristics		All samples (n = 112)		Treatment arm						GWMS				
				CETU/IRI* (n = 58)		IRI* (n = 54)		p		HMCC** (n = 47)		LMCC** (n = 65)		p
Gender	Male	62	55.4	30	51.7	32	59.3	0.42^+^		26	55.3	36	55.4	0.99^+^
No. (%)	Female	50	44.6	28	48.3	22	40.7			21	44.7	29	44.6	
Median age, years (range)		61.5 (29—85)		59.5 (34—85)		62.5 (29—82)		0.56^++^		65.0 (29—85)		59.0 (35—80)		0.40^++^
Primary tumor site	Colon	97	86.7	50	86.2	47	87.0	0.90^+^		44	93.6	53	81.5	0.09^+++^
No. (%)	Rectum	15	13.3	8	13.8	7	13.0			3	6.4	12	18.5	
Race	White	80	71.4	38	65.5	42	77.8	0.15^+^		35	74.5	45	69.2	0.54^+^
No. (%)	Others	32	28.6	20	34.5	12	22.2			12	25.6	20	30.8	
PS	0	57	50.9	31	53.4	26	48.1	0.71^+++^		24	51.1	33	50.8	0.88^+++^
No. (%)	1	49	43.8	25	43.1	24	44.4			20	42.6	29	44.6	
	2	5	4.5	2	3.4	3	5.6			2	4.3	3	4.6	
	NA	1	0.8	0	0	1	1.9			1	2.1	0	0	
Assigned treatment	CETU + IRI	58	51.8	58	100	0	0	n/a		24	51.1	34	52.3	0.90^+^
No. (%)	IRI	54	48.2	0	0	54	100			23	48.9	31	47.7	
GWMS	HMCC	47	42.0	24	41.4	23	42.6	0.90^+^		47	100	0	0	n/a
No. (%)	LMCC	65	58.0	34	58.6	31	57.4			0	0	65	100	
Liver metastasis	Yes	80	71.4	43	74.1	37	68.5	0.51^+^		33	70.2	47	72.3	0.84^+^
No. (%)	No	30	26.8	14	24.1	16	29.6			13	27.7	17	26.2	
	NA	2	1.8	1	1.7	1	1.9			1	2.1	1	2.1	

^*^CETU and IRI denotes Cetuximab and Irinotecan, respectively. **HMCC and LMCC denotes high-methylated and low-methylated colorectal cancer, respectively

GWMS: genome wide DNA methylation status, PS: performance status, n/a: not available

^+^X2 test ^++^Wilcoxon test, ^+++^Fisher’s exact test

### Genome-wide DNA methylation status (GWMS)

DNA methylation was measured using the modified MethyLight assay for the 16 CpG sites, with a median methylation positive rate for each CpG site of 42.9% (12.5%–73.2%) (Supplementary Fig. 2). Based on the number of methylation-positive sites, the GWMS of each case was determined to be HMCC in 47 patients (42.0%) and LMCC in 65 patients (58.0%) (Table 1, Supplementary Fig. 2).

### Comparison of patient backgrounds

Patient backgrounds were compared between the two groups with different treatment arms (CETU/IRI vs. IRI) or with different GWMS (HMCC vs. LMCC), respectively (Table 1). We found no significant differences in any of the baseline patient characteristics between the two treatment arms. Comparisons focusing on GWMS showed a trend toward a higher rate of rectal cancer in the LMCC group, whereas comparisons of other parameters revealed no significant differences between the two groups.

### Differences in clinical outcomes between the GWMS subgroups

To clarify the impact of the differences in GWMS on clinical outcomes, OS, PFS, and RR were compared between the HMCC and LMCC groups in each treatment arm.

**Fig. 1:**
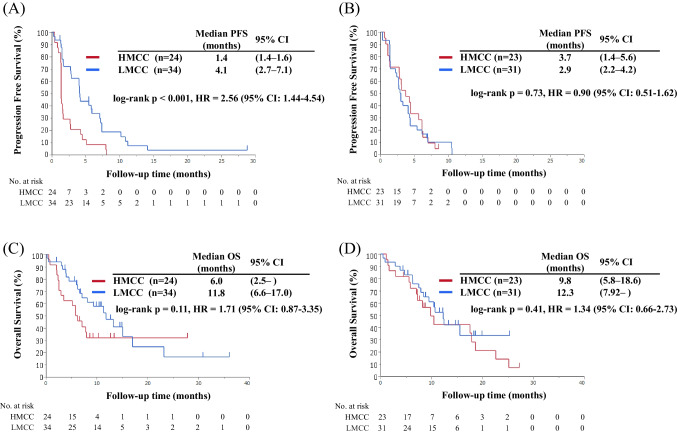
Comparison of survival times between the HMCC and the LMCC groups in each treatment arm. Panels (A) and (C) demonstrate the progression-free survival (PFS) and overall survival (OS) in the cetuximab plus irinotecan arm of the HMCC group (red line, n = 24) and the LMCC group (blue line, n = 34). Panels (B) and (D) depict the PFS and OS in the irinotecan arm of the HMCC group (red line, n = 23) and the LMCC group (blue line, n = 31). The survival curves were generated using the Kaplan–Meier method, and the differences were assessed using the log-rank test. Abbreviations: PFS, progression-free survival; OS, overall survival; CI, confidence interval; HMCC, high-methylated colorectal cancer; LMCC, low-methylated colorectal cancer

In the CETU/IRI arm, the median PFS of the HMCC group was significantly shorter than that of the LMCC group (median PFS: 1.4 vs. 4.1 months, p < 0.001, HR = 2.56, 95% CI: 1.44–4.54; Fig. 1A). Moreover, the OS of the HMCC group was numerically shorter than that of the LMCC group, although the difference was nonsignificant (median OS: 6.0 vs. 11.8 months, p = 0.11, HR = 1.71, 95% CI: 0.87–3.35; Fig. 1C). The RR of the HMCC group was significantly lower than that of the LMCC group (0% vs. 26.6%, p = 0.02; Table 2).

**Table 2:** Best overall response for each GWMS

	CETU/IRI							IRI						
Best overall response														
	All samples (n = 58)			HMCC (n = 24)	LMCC (n = 34)		p	All samples (n = 54)		HMCC (n = 23)		LMCC (n = 31)		p
	**n**	**%**	**n**	**%**	**n**	**%**		**n**	**%**	**n**	**%**	**n**	**%**	
RR (%)		16.0		0		26.6	0.02^+^		8.7		5.6		10.7	0.63^+^
CR	1	2.0	0	0	1	3.3		0	0	0	0	0	0	
PR	7	14.0	0	0	7	23.3		4	8.7	1	5.6	3	10.7	
SD	21	42.0	7	35.0	14	46.7		27	58.7	12	66.7	15	53.6	
PD	21	42.0	13	65.0	8	26.7		15	32.6	5	27.8	10	35.7	
NE	8		4		4			8		5		3		

CR, complete response, PR, partial response: SD, stable disease: PD, progressive disease: RR, response rate. ^+^Fisher’s exact test

In the IRI arm, there was no significant difference between the HMCC and LMCC groups in PFS, OS, and RR (median PFS: 3.7 vs. 2.9 months, p = 0.73, HR = 0.90, 95% CI: 0.51–1.62; median OS: 9.8 vs. 12.3 months, p = 0.41, HR = 1.34, 95% CI: 0.66–2.73; RR: 5.6% vs. 10.7%, p = 0.63, respectively; Figs. 1B, D and Table 2).

### Differences in clinical outcomes between the treatment arms

To determine the impact of the assigned treatment arm on clinical outcomes, PFS, OS, and RR were compared between the CETU/IRI and IRI arms. In total cohort (n = 112), there were no significant differences in PFS, OS, and RR between treatment arms (Supplementary Fig. 3, Supplementary Table 1).

**Fig. 2:**
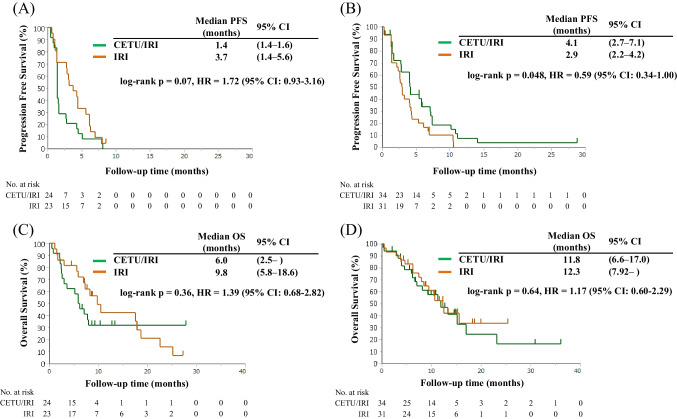
Comparison of survival times between the CETU/IRI arm and the IRI arm in each GWMS. Panels (A) and (C) show the progression-free survival (PFS) and overall survival (OS) in patients with HMCC in the CETU/IRI arm (green line, n = 24) and the IRI arm (yellow line, n = 23). Panels (B) and (D) depict the PFS and OS in patients with LMCC in the CETU/IRI arm (green line, n = 34), and the IRI arm (yellow line, n = 31). The survival curves were generated using the Kaplan–Meier method, and the differences were assessed using the log-rank test. Abbreviations: PFS, progression-free survival; OS, overall survival; CI, confidence interval; CETU, cetuximab; IRI irinotecan; HMCC, high-methylated colorectal cancer; LMCC, low-methylated colorectal cancer

Next, clinical outcomes were compared between the CETU/IRI and IRI arms in each GWMS group. The PFS and OS of the HMCC group were numerically shorter in the CETU/IRI arm vs. the IRI arm, although the difference was statistically nonsignificant (median PFS: 1.4 vs. 3.7 months, p = 0.07, HR = 1.72, 95% CI: 0.93–3.16; median OS: 6.0 vs. 9.8 months, p = 0.36, HR = 1.39, 95% CI: 0.68–2.82, respectively; Figs. 2A, C). In addition, the RR of the HMCC group was not significantly different between the two arms (0% vs. 5.6%, p = 0.49; Supplementary Table 1).

The PFS of the LMCC group was significantly longer in the CETU/IRI arm than that of the IRI arm (median PFS: 4.1 vs. 2.9 months, p = 0.048, HR = 0.59, 95% CI: 0.34–1.00; Fig. 2B). The OS of the LMCC group was not significantly different between the two arms (median OS: 11.8 vs. 12.3 months, p = 0.64, HR = 1.17, 95% CI: 0.60–2.29; Fig. 2D). Finally, the RR of the LMCC group showed a trend toward higher values in the CETU/IRI arm vs. the IRI arm, although the difference was nonsignificant (26.6% vs. 10.7%, p = 0.19; Supplementary Table 1).

### Factors contributing to the PFS and OS in each treatment arm

Univariate and multivariate analyses were performed using patient background parameters, including GWMS, to identify factors that contributed to or confounded the PFS and OS in each treatment arm.

**Fig. 3:**
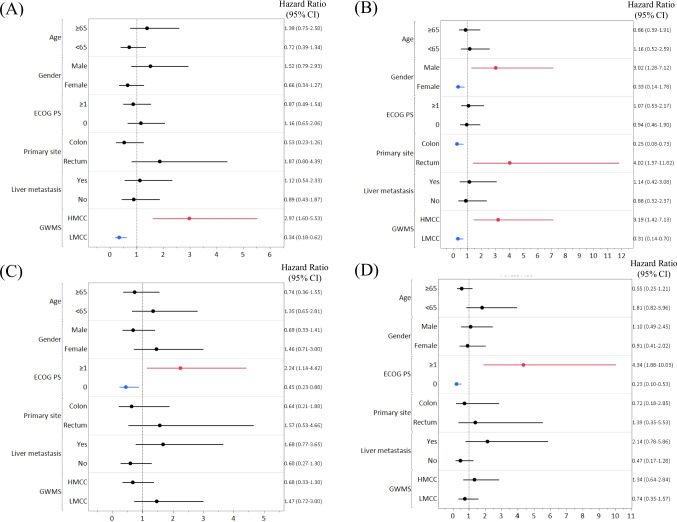
Forest plot for HR of PFS and OS. Panels (A) and (B) depict the HR of PFS and OS in the CETU/IRI arm. Panels (C) and (D) depict the HR of PFS and OS in the IRI arm. The red line indicates significant poor prognostic factors; the blue line indicates significant favorable prognostic factors. Abbreviations: HR, hazard ratio; PFS, progression-free survival; OS, overall survival; CI, confidence interval; CETU, cetuximab; IRI irinotecan; HMCC, high-methylated colorectal cancer; LMCC, low-methylated colorectal cancer; ECOG PS, Eastern Cooperative Oncology Group Performance Status

In the multivariate analysis, only GWMS (p = 0.002) was an independent predictor of PFS in the CETU/IRI arm; in turn, not only GWMS (p = 0.005) but also gender (p = 0.012) and primary tumor site (p = 0.011) were identified as independent prognostic factors (Figs. 3A, B and Supplementary Table 2).

In the multivariate analysis of PFS and OS in the IRI arm, neither the primary tumor site nor GWMS were significant predictors of either PFS or OS; PS alone was extracted as an independent predictor (p = 0.020, p < 0.001, respectively; Figs. 3C, D and Supplementary Table 3).

Tests for interaction were conducted between GWMS (HMCC or LMCC) and anti-EGFR combination (CETU/IRI or IRI). As a result, a significant interaction was observed between the subgroups for PFS (p for interaction = 0.006), but no significant interaction was observed for OS (p for interaction = 0.613).

## Discussion

This study examined the effect of the genome-wide DNA methylation status on the efficacy of the addition of an anti-EGFR antibody to irinotecan in the second-line treatment of mCRC. We verified the significance of GWMS as a predictive factor of clinical outcomes in patients treated with anti-EGFR antibodies. To confirm whether a novel biomarker predicts the response to a given treatment, it is necessary to compare its predictive performance between groups that received the treatment and those that did not receive it. However, it is difficult to conduct new clinical trials comparing treatment efficacy with or without anti-EGFR antibodies because the significance of anti-EGFR antibodies has been well-established in the treatment of mCRC. In this translational research, such analysis was possible via the use of clinical data and tumor specimens collected in a previous large-scale prospective clinical trial. This study was able to evaluate the impact of GWMS on the effect of anti-EGFR antibodies more rigorously than previous reports [26, 27] because the treatment line and combination therapy were standardized. On the other hand, it should be noted that this study included second-line treatment patients who had not received irinotecan, so differences in the therapeutic effect of irinotecan may have affected the results.

Diagnostic assays that use gDNA extracted from tumor specimens sometimes encounter problems regarding the long-term preservation of samples after FFPE embedding. The EPIC study was conducted between 2003 and 2006, and the FFPE specimens used for DNA extraction were all fixed more than 15 years ago. Nevertheless, the GWMS could be measured in the present study in all patients from which a sufficient amount of gDNA was extracted. These facts suggest that our modified MethyLight assay is not affected by the duration of the storage of the specimens.

Although previous studies investigating the association between GWMS and the efficacy of anti-EGFR antibodies have focused on Japanese patients [26–29], the present study was performed on Western patients with colorectal cancer. In this study, about 40% of patients were classified as HMCC, which was higher than previously reported [27, 28], suggesting that a higher percentage of Western patients with colorectal cancer have HMCC compared with Japanese patients. However, detailed information on the primary tumor site and *BRAF* genotype, which could be related to the distribution of GWMS, is needed to establish a rigorous comparison with previous reports. In a comparison of patient backgrounds, LMCC exhibited a trend toward a higher proportion among patients with rectal cancer compared with HMCC. This result was consistent with previous studies reporting that hypermethylated colorectal cancer tends to occur in the right-sided colon [23, 27, 28, 30].

The LMCC group showed a trend toward better clinical outcomes compared with the HMCC group in the CETU/IRI arm. Conversely, there was no significant difference in clinical outcome between the two groups in the IRI arm. These results supported the significance of GWMS not only as a prognostic factor in mCRC but also as a predictive factor of treatment response to anti-EGFR antibodies. The analytical results obtained in the IRI arm were consistent with previous reports that GWMS is not associated with the therapeutic efficacy of cytotoxic agents [29, 31]. In multivariate analyses, GWMS was extracted as an independent predictor of both PFS and OS in the CETU/IRI arm, whereas only PS was extracted as an independent predictor of both outcomes in the IRI arm. These findings also support the opinion that GWMS is a predictive factor of response to anti-EGFR antibodies rather than a prognostic factor.

The comparison of clinical outcomes between the treatment arms (CETU/IRI vs. IRI) for each GWMS group showed that only LMCC exhibited a significant prolongation of PFS with the addition of anti-EGFR antibodies, whereas HMCC showed no survival benefit with the addition of these agents. These results validate the hypothesis that LMCC is sensitive to anti-EGFR antibodies, whereas HMCC is refractory to them. Regarding OS, no significant difference in survival time was observed in the LMCC group with or without concomitant anti-EGFR antibodies. This result may be attributed to the post-study therapy with anti-EGFR antibodies, which was allowed in the IRI arm; 47% of patients assigned to the IRI arm went on to receive cetuximab post-study with 87% of those, receiving cetuximab in combination with irinotecan, according to a previous report [9]. Notably, HMCC exhibited a trend toward a numerically shorter OS and PFS in the CETU/IRI arm compared with the IRI arm, although the difference was statistically nonsignificant. These results suggest a detrimental effect of anti-EGFR antibody treatment in HMCC as well as in patients with *KRAS* mutations [32]. Moreover, these findings further emphasize the importance of GWMS in selecting patients with indications for treatment with anti-EGFR antibodies.

There have been several reports on the mechanisms underlying how aberrant DNA methylation affects the sensitivity to anti-EGFR antibodies for mCRC. Lee et al*.* reported that aberrant DNA methylation in the promoter region of *AREG*/*EREG* defined sensitivity to anti-EGFR antibodies through regulation of gene expression [30]. Otsuki et al*.* reported that the expression status of a set of genes (the cetuximab signature [33]) associated with anti-EGFR antibodies sensitivity was regulated by aberrant DNA methylation [34]. The mechanism underlying the association between aberrant DNA methylation and the clinical outcomes of anti-EGFR antibodies is not fully understood and requires further investigation.

This study had some limitations. First, the final analysis of this study was conducted on a significantly small subset (n = 112) of patients enrolled in the EPIC trial (n = 1,298). The EPIC trial was conducted more than a decade ago, and the lack of tumor tissue for DNA extraction and clinical data limited the number of patients for whom DNA methylation analysis was possible. This study population had a higher proportion of females (44.6% vs. 37.1%) and a lower proportion of whites (71.4% vs. 90.9%) compared to the ITT population in the EPIC trial [9]. Previous reports had suggested that females may receive less benefit from anti-EGFR antibodies than male [35], which might lead to the fact that the present cohort did not show the prolonged PFS in the CETU/IRI arm (Supplementary Fig. 3) that was observed in the ITT population of the EPIC trial [9]. Furthermore, race indicated the possibility of different genetic mutation profiles related to anti-EGFR antibody sensitivity in mCRC [36]. Therefore, the difference in the proportion of race between the small cohort in this study and the ITT population of the EPIC trial might have had some influence on the outcomes. However, as shown in Table 1, there were no significant gender and race biases among treatment groups or GWMS, suggesting that these factors had little impact on the results.

Second, the information on the *RAS/BRAF* genotype and microsatellite instability (MSI) were lacking, which were important gene alterations in mCRC that affects the sensitivity to anti-EGFR antibodies [14–16, 37]. Previous reports have shown that almost all colorectal cancers with *BRAF* mutation are classified as HMCC [27, 29, 38]; therefore, the inclusion of patients with *BRAF* mutation may have contributed to the poor clinical outcomes observed in the HMCC group. *RAS* mutations were detected in both HMCC and LMCC, with no significant preference between the two groups in previous reports [27, 29, 38]. Importantly, it has been shown that *RAS/BRAF* wild-type HMCC may be as resistant to anti-EGFR antibodies as mCRC with *RAS* mutation [27, 29]. Thus, patients with *RAS* mutation may have little impact on the clinical outcomes of anti-EGFR antibody therapy in the HMCC group. Conversely, the presence of *RAS* mutant in patients with mCRC including LMCC may have led to even worsening clinical outcomes in the LMCC group treated with anti-EGFR antibody. Therefore, we believe that the presence of the potential *RAS* mutant in patients with mCRC does not negate the findings of this study. Nevertheless, because the *RAS/BRAF* genotype is an essential factor in the evaluation of the therapeutic efficacy of anti-EGFR antibodies and the coexistence of driver mutations such as *TP53* gain-of-function mutations along with HMCC being associated with the poor prognosis of mCRC [38], the findings of this study need to be validated in a larger group of patients with wild-type *RAS/BRAF*. It is known that HMCC contains a higher proportion of mismatch repair deficient (MMR-D) patients than LMCC [38]. MMR-D patients have been reported to be resistant to anti-EGFR antibodies, which may contribute to worse clinical outcomes of HMCC. Furthermore, other genetic alterations (*PTEN*, *EGFR* ECD exons 1–16, amplifications of *HER2* and *MET*, gene fusions of *RET*, *NRTK1,* and *ALK*) reported to be associated with sensitivity of anti-EGFR antibodies [39] were not examined in this study and may have had some influence on the results.

Third, this study did not include detailed information on the primary tumor site. Sidedness, which has become important in recent years, is often used to classify mCRC into right-sided colon cancer and left-sided colon/rectal cancer, according to the splenic flexure [40, 41]. Recent evidence suggests that patients with left-sided colorectal cancer are associated with favorable clinical benefits from anti-EGFR antibodies as a first-line treatment, and that those with right-sided colorectal cancer are associated with worse clinical benefits [41]. However, the present study provided information regarding whether the primary tumor site was colon or rectum, not the sidedness. Therefore, in this study, it remains unclear what to extent the sidedness affects the predictive value of GWMS for anti-EGFR antibody. Of note, several previous reports exploring the relationship between the sidedness and GWMS in predicting the clinical outcomes of anti-EGFR antibodies have shown that GWMS is a predictor of therapeutic effects independent of the sidedness [27, 29]. There are also reports that there is a little association between the clinical outcomes of anti-EGFR antibodies and sidedness in second- or later-line treatment [42]. Thus, GWMS may be associated with clinical outcomes of anti-EGFR antibodies as second line treatment, regardless of the sidedness.

## Conclusions

GWMS measured using the modified MethyLight assay is a predictive factor of the clinical outcomes of anti-EGFR antibody therapy in the second-line treatment of metastatic colorectal cancer.

## Autor contributions

Kota Ouchi: study design, data acquisition, quality control of data and algorithms, data analysis and interpretation, statistical analysis, manuscript preparation, editing, and review; Shin Takahashi: study design, quality control of data and algorithms, data analysis and interpretation, statistical analysis, manuscript review; Chikashi Ishioka: study concepts, study design, data analysis and interpretation, manuscript preparation, editing, and review; Keiju Sasaki, Yuya Yoshida, Sakura Taniguchi, Yuki Kasahara, Keigo Komine, Hiroo Imai, Ken Saijo, Hidekazu Shirota, Masanobu Takahashi: data acquisition and interpretation, manuscript review.

## Supplementary Information

Below is the link to the electronic supplementary material.

Supplementary file1 (DOCX 41.1 KB)

Supplementary file2 (PPTX 127 KB)

## Data Availability

The data generated in this study are available upon request from the corresponding author.

## Abbreviations

GWMS: Genome-wide DNA methylation status
EGFR: Epidermal growth factor receptor
mCRC: Metastatic colorectal cancer
HMCC: High-methylated colorectal cancer
LMCC: Low-methylated colorectal cancer
PFS: Progression-free survival
OS: Overall survival
CIMP: CpG island methylator phenotype
FFPE: Formalin-fixed, paraffin-embedded
CET: Cetuximab
IRI: Irinotecan
BOR: Best overall response
RR: Response rate
ECOG PS: Eastern Cooperative Oncology Group Performance Status
gDNA: Genomic DNA
