# Supplementary material for: Genome-wide DNA methylation status is a predictor of the efficacy of anti-EGFR antibodies in the second-line treatment of metastatic colorectal cancer: Translational research of the EPIC trial
Source: Int J Colorectal Dis. 2024 Jun 11;39(1):89. doi: 10.1007/s00384-024-04659-y (PMC11166830; doi:10.1007/s00384-024-04659-y)
Supplement: Supplementary file 1 — Supplementary file1 (DOCX 41.1 KB) [file 384_2024_4659_MOESM1_ESM.docx]

**Supplementary Tables**

| **Supplementary Table 1. Best overall response for each treatment arm** | | | | | | | | | | | | | | | | | | | | | | | | | | | | | | | | | | | |
| --- | --- | --- | --- | --- | --- | --- | --- | --- | --- | --- | --- | --- | --- | --- | --- | --- | --- | --- | --- | --- | --- | --- | --- | --- | --- | --- | --- | --- | --- | --- | --- | --- | --- | --- | --- |
|  |  | **Total cohort** | | | | | | | | | |  | **HMCC** | | | | | | | | | | |  | **LMCC** | | | | | | | | | | |
| **Best overall response** |  |  |  |  |  |  |  |  |  |  |  |  |  |  |  |  |  |  |  |  |  |  |  |  |  |  |  |  |  |  |  |  |  |  |  |
|  |  | **All samples**  **(n = 112)** | |  | **CETU/IRI**  **(n = 58)** | |  | **IRI**  **(n = 54)** | |  | **p** |  | **All samples**  **(n = 47)** | |  | **CETU/IRI**  **(n = 24)** | |  | **IRI**  **(n = 23)** | |  | **p** | |  | **All samples**  **(n = 65)** | |  | **CETU/IRI**  **(n = 34)** | |  | **IRI**  **(n = 31)** | |  | **p** | |
|  |  | **n** | **%** |  | **N** | **%** |  | **n** | **%** |  |  |  | **n** | **%** |  | **n** | **%** |  | **n** | **%** |  |  |  |  | **n** | **%** |  | **n** | **%** |  | **n** | **%** |  |  |  |
| RR (%) |  |  | 12.5 |  |  | 16.0 |  |  | 8.7 |  | 0.36^+^ |  |  | 2.6 |  |  | 0 |  |  | 5.6 |  | 0.49^+^ | |  |  | 18.9 |  |  | 26.6 |  |  | 10.7 |  | 0.19^+^ | |
| CR |  | 1 | 1.0 |  | 1 | 2.0 |  | 0 | 0 |  |  |  | 0 | 0 |  | 0 | 0 |  | 0 | 0 |  |  |  |  | 1 | 1.7 |  | 1 | 3.3 |  | 0 | 0 |  |  |  |
| PR |  | 11 | 11.5 |  | 7 | 14.0 |  | 4 | 8.7 |  |  |  | 1 | 2.6 |  | 0 | 0 |  | 1 | 5.6 |  |  |  |  | 10 | 17.2 |  | 7 | 23.3 |  | 3 | 10.7 |  |  |  |
| SD |  | 48 | 50.0 |  | 21 | 42.0 |  | 27 | 58.7 |  |  |  | 19 | 50.0 |  | 7 | 35.0 |  | 12 | 66.7 |  |  |  |  | 29 | 50.0 |  | 14 | 46.7 |  | 15 | 53.6 |  |  |  |
| PD |  | 36 | 37.5 |  | 21 | 42.0 |  | 15 | 32.6 |  |  |  | 18 | 47.4 |  | 13 | 65.0 |  | 5 | 27.8 |  |  |  |  | 18 | 31.0 |  | 8 | 26.7 |  | 10 | 35.7 |  |  |  |
| NE |  | 16 |  |  | 8 |  |  | 8 |  |  |  |  | 9 |  |  | 4 |  |  | 5 |  |  |  |  |  | 7 |  |  | 4 |  |  | 3 |  |  |  |  |
| CR, complete response, PR, partial response: SD, stable disease: PD, progressive disease: RR, response rate. ^+^Fisher’s exact test. | | | | | | | | | | | | | | | | | | | | | | | | | | | | | | | | | | | |

| **Supplementary Table 2.**  **Univariate and multivariate analyses of PFS and OS for the CETU/IRI arm (n = 58)** | | | | | | | | |
| --- | --- | --- | --- | --- | --- | --- | --- | --- |
|  | **PFS** | | | | **OS** | | | |
|  | Univariate | | Multivariate | | Univariate | | Multivariate | |
|  | HR |  | HR |  | HR |  | HR |  |
| Variable | (95% CI) | p | (95% CI) | p | (95% CI) | p | (95% CI) | p |
| Age  （≥65 vs. <65） | 1.68 | 0.08 | 1.38 | 0.30 | 1.19 | 0.63 | 0.86 | 0.71 |
|  | (0.94–3.00) |  | (0.75-2.58) |  | (0.59–2.42) |  | (0.39-1.91) |  |
| Gender  （Male vs. Female） | 1.34 | 0.29 | 1.52 | 0.21 | 2.38 | 0.014 | 3.02 | 0.012 |
|  | (0.78–2.31) |  | (0.79-2.93 |  | (1.19–4.75) |  | (1.28–7.12) |  |
| ECOG PS  (0 vs. ≥1) | 1.12 | 0.69 | 1.16 | 0.63 | 1.03 | 0.93 | 0.94 | 0.86 |
|  | (0.65-1.93) |  | (0.65-2.06) |  | (0.53-2.01) |  | (0.46-1.90) |  |
| Primary tumor site　 (Colon vs. Rectum） | 0.76 | 0.50 | 0.53 | 0.15 | 0.53 | 0.20 | 0.25 | 0.011 |
|  | (0.34–1.69) |  | (0.23-1.26) |  | (0.20–1.39) |  | (0.08–0.73) |  |
| Liver metastasis  (Yes vs. No) | 1.24 | 0.51 | 1.12 | 0.77 | 1.66 | 0.23 | 1.14 | 0.80 |
|  | (0.66–2.34) |  | (0.54-2.33) |  | (0.72–3.83) |  | (0.42-3.08) |  |
| GWMS  (HMCC vs. LMCC) | 2.56 | 0.001 | 2.97 | <0.001 | 1.71 | 0.12 | 3.19 | 0.005 |
|  | (1.44–4.54) |  | (1.60-5.53) |  | (0.87–3.35) |  | (1.42–7.14) |  |
| PFS = progression-free survival; OS = overall survival; HR = hazard ratio; CI = confidence interval | | | | | | | | |

| **Supplementary Table 3. Univariate and multivariate analyses of PFS and OS for the IRI arm (n = 54)** | | | | | | | | |
| --- | --- | --- | --- | --- | --- | --- | --- | --- |
|  | **PFS** | | | | **OS** | | | |
|  | Univariate | | Multivariate | | Univariate | | Multivariate | |
|  | HR |  | HR |  | HR |  | HR |  |
| Variable | (95% CI) | p | (95% CI) | p | (95% CI) | p | (95% CI) | p |
| Age  （≥65 vs. <65） | 1.07 | 0.81 | 0.74 | 0.42 | 0.68 | 0.29 | 0.55 | 0.14 |
|  | (0.60–1.91) |  | (0.36-1.55) |  | (0.34–1.38) |  | (0.25-1.21) |  |
| Gender  （Male vs. Female） | 0.91 | 0.75 | 0.69 | 0.31 | 1.12 | 0.75 | 1.10 | 0.82 |
|  | (0.51–1.62) |  | (0.33-1.41) |  | (0.56–2.26) |  | (0.49-2.45) |  |
| ECOG PS  (0 vs. ≥1) | 0.55 | 0.046 | 0.45 | 0.020 | 0.28 | 0.001 | 0.23 | <0.001 |
|  | (0.31-0.99) |  | (0.23-0.88) |  | (0.13-0.61) |  | (0.10-0.53) |  |
| Primary tumor site　 (Colon vs. Rectum） | 0.81 | 0.63 | 0.64 | 0.41 | 1.05 | 0.93 | 0.72 | 0.64 |
|  | (0.34–1.92) |  | (0.21-1.88) |  | (0.36–3.03) |  | (0.18–2.85) |  |
| Liver metastasis  (Yes vs. No) | 1.06 | 0.85 | 1.68 | 0.19 | 1.40 | 0.41 | 2.14 | 0.14 |
|  | (0.57–1.99) |  | (0.77-3.65) |  | (0.63–3.12) |  | (0.78-5.86) |  |
| GWMS  (HMCC vs. LMCC) | 0.90 | 0.74 | 0.68 | 0.29 | 1.34 | 0.41 | 1.34 | 0.44 |
|  | (0.51–1.62) |  | (0.33-1.38) |  | (0.66–2.73) |  | (0.64–2.84) |  |
| PFS = progression-free survival; OS = overall survival; HR = hazard ratio; CI = confidence interval | | | | | | | | |
